# P300 acetyltransferase regulates fatty acid synthase expression, lipid metabolism and prostate cancer growth

**DOI:** 10.18632/oncotarget.7715

**Published:** 2016-02-25

**Authors:** Xiaokun Gang, Yinhui Yang, Jian Zhong, Kui Jiang, Yunqian Pan, R. Jeffrey Karnes, Jun Zhang, Wanhai Xu, Guixia Wang, Haojie Huang

**Affiliations:** ^1^ Department of Endocrinology and Metabolism, The First Hospital of Jilin University, Changchun, Jilin 130021, China; ^2^ Department of Biochemistry and Molecular Biology, Mayo Clinic College of Medicine, Rochester, MN 55905, USA; ^3^ Department of Urology, The Fourth Hospital of Harbin Medical University, Harbin, Heilongjiang 150001, China; ^4^ Department of Oncology, The Second affiliated Hospital of Dalian Medical University, Dalian, Liaoning 116027, China; ^5^ Department of Urology, Mayo Clinic College of Medicine, Rochester, MN 55905, USA; ^6^ Department of Laboratory Medicine and Pathology, Mayo Clinic College of Medicine, Rochester, MN 55905, USA; ^7^ Mayo Clinic Cancer Center, Mayo Clinic College of Medicine, Rochester, MN 55905, USA

**Keywords:** P300, FASN, histone acetylation, lipid metabolism, prostate cancer

## Abstract

*De novo* fatty acid (FA) synthesis is required for prostate cancer (PCa) survival and progression. As a key enzyme for FA synthesis fatty acid synthase (FASN) is often overexpressed in human prostate cancers and its expression correlates with worse prognosis and poor survival. P300 is an acetyltransferase that acts as a transcription co-activator. Increasing evidence suggests that P300 is a major PCa promoter, although the underlying mechanism remains poorly understood. Here, we demonstrated that P300 binds to and increases histone H3 lysine 27 acetylation (H3K27Ac) in the *FASN* gene promoter. We provided evidence that P300 transcriptionally upregulates *FASN* expression and promotes lipid accumulation in human PCa cells in culture and *Pten* knockout prostate tumors in mice. Pharmacological inhibition of P300 decreased FASN expression and lipid droplet accumulation in PCa cells. Immunohistochemistry analysis revealed that expression of P300 protein positively correlates with FASN protein levels in a cohort of human PCa specimens. We further showed that FASN is a key mediator of P300-induced growth of PCa cells in culture and in mice. Together, our findings demonstrate P300 as a key factor that regulates FASN expression, lipid accumulation and cell growth in PCa. They also suggest that this regulatory pathway can serve as a new therapeutic target for PCa treatment.

## INTRODUCTION

Prostate cancer (PCa) is one of the most commonly diagnosed male malignancies in the United States. PCa accounts for approximately 19% of the new cases each year, and up to 4% of patients have metastases at the time of diagnosis [1, 2]. Aberrant cellular metabolism is a core hallmark of cancer [3]. The highly proliferative nature of cancerous cells imposes a large requirement for biosynthesis of amino acids, nucleic acids and lipids. Cancer cells generally exhibit increased conversion of glucose to lactate by aerobic glycolysis, a phenomenon termed Warburg effect. However, it does not seem the case in primary prostate cancers since PCa cannot be effectively detected by ^18^F-deoxyglucose positron emission tomography (^18^F-FDG-PET). Instead, due to the fact that choline is an essential component of the cell membrane and choline kinase activity is upregulated in PCa [4], ^11^C-choline PET is superior to ^18^F-FDG-PET for PCa imaging [5] and is approved by US FDA for detection of recurrent PCa. Accordingly, increased *de novo* lipid synthesis is often detected in PCa where overexpression of lipogenic enzymes such as FASN occurs in both early (prostate intraepithelial neoplasia (PIN)) and late (metastasis) stages of PCa [6-8]. Transgenic animal studies demonstrate that *FASN* is a *bona fide* oncogene in PCa [9]. Thus, fatty acid metabolism has become a potential focus for treatment of PCa.

FASN is a key enzyme for *de novo* fatty acid (FA) synthesis. It is a 270-kDa enzyme that forms a dimer in cytoplasm, which can process one acetyl-CoA and seven malonyl-CoA molecules to produce palmitate and other long-chain FA. Expression and activity of FASN are regulated by growth factors, hormones and dietary factors [10]. FASN expression has been shown to be upregulated in early stage of PCa and increased during disease progression [11]. High expression of FASN also associates with poor prognosis and inhibition of FASN results in cancer cell death and reduction in tumor volume [12, 13]. The regulation of FASN expression appears to be very complicated. It occurs at both transcriptional and post-transcriptional levels. However, the precise mechanism underlying FASN expression is not fully understood.

P300, also known as EP300 (E1A binding protein P300), is an essential co-activator in gene transcription control. The main function modules in this protein consist of: (a) bridging DNA binding factors and general transcription factors; (b) catalyzing histone acetylation via its intrinsic histone acetyltransferase activity; and (c) acetylating transcriptional factors to further facilitate their activity. Through these various mechanisms, P300 is involved in the regulation of expression and function of a large number of tumor-relevant proteins, including oncoproteins c-Myc [14], CREB [15] and androgen receptor (AR) [16] and tumor suppresser proteins p53 [17] and breast cancer gene-1, BRCA1 [18]. Therefore, P300 is a double-edged sword for tumor growth depending on the cell types and the associated signaling pathways. The previous studies consistently show that P300 is overexpressed in human PCa and P300 overexpression promotes proliferation of PCa cells in culture and in mice and its expression associates with human PCa progression [16, 19, 20]. These findings suggest that P300 is a major promoter of PCa, although the underlying mechanism remains elusive.

In the present study, we found that P300 binds to the *FASN* gene promoter and transcriptionally activates *FASN* gene expression in PCa cells. We also showed that P300 induced FA synthesis and lipid droplet accumulation in PCa cells both *in vitro* and *in vivo*. We found that P300 protein expression positively correlates with FASN protein levels in human PCa specimens and demonstrated that FASN is a key downstream mediator of P300-induced PCa cell growth *in vitro* and *in mice*.

## RESULTS

### P300 binds to and regulates H3K27Ac in the *FASN* gene promoter in PCa cells

**Figure 1:**
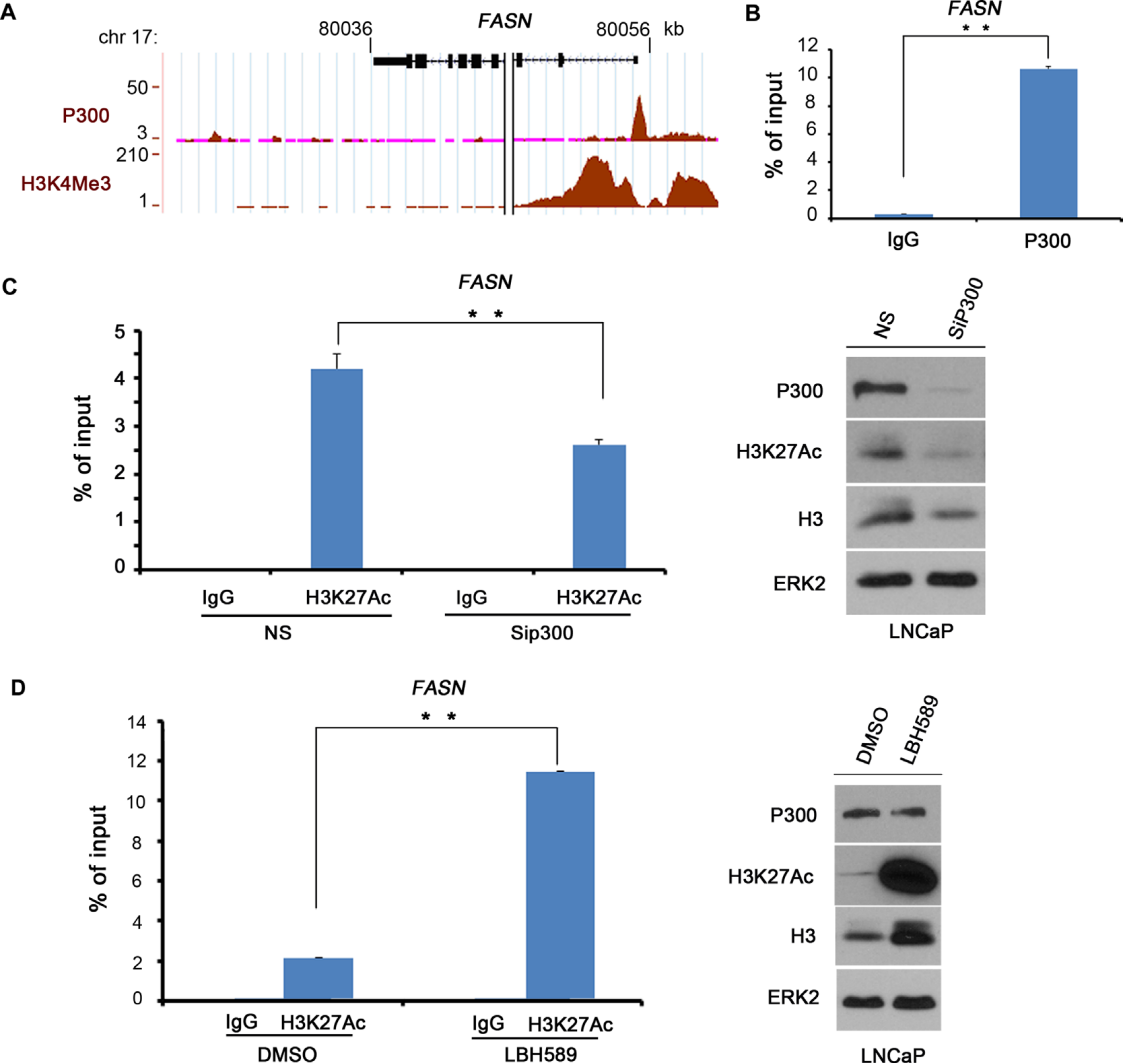
P300 binds to the *FASN* gene promoter **A.** Screen shots from the UCSC genome browser showing signal profiles of P300 binding (ChIP-seq) and H3K4Me3 modifications (ChIP-seq), a histone mark of promoters as reported previously by others in LNCaP cells [46]. **B.** P300 binds to the *FASN* gene promoter as demonstrated by ChIP assay. LNCaP cells were cultured in 10% FBS medium for 48 h and cell lysates were subject to ChIP assay using control IgG or P300 antibody. Immunoprecipitated DNA was analyzed by real-time PCR using the primers in the predicted P300 binding region in the *FASN* gene promoter. **C.** P300 knockdown affects H3K27Ac levels at the *FASN* promoter. LNCaP cells were transfected with non-specific (NS) control or P300-specific siRNAs for 48 h prior to ChIP assay with control IgG or H3K27Ac antibody and western blot analysis with indicated antibodies. **D.** The HDAC inhibitor treatment increases H3K27Ac levels at the *FASN* promoter. C4-2 cells were treated with DMSO or Panobinostat (LBH589) at 20 nM for 24 h prior to ChIP assay and western blot analysis with indicated antibodies. Columns, mean values among three replicates; error bars, SD. ** *P* < 0.01.

FASN is a key enzyme that regulates FA metabolism and plays an important role in the energy balance in cancer cells. It is found overexpressed in PCa. P300 is a major transcription co-activator that promotes PCa growth and progression. We sought to determine whether P300 regulates *FASN* gene expression in PCa cells. Meta-analysis of P300 ChIP-seq data in the public domain showed that there is an obvious binding peak near the transcription start site (TSS) at the promoter of the *FASN* gene in LNCaP PCa cells (Figure 1A). The authenticity of the promoter is evident by the enrichment of the histone modification H3 lysine 4 trimethylation (H3K4Me3) [29]. We performed a CHIP assay to confirm the binding of P300 at the *FASN* promoter in LNCaP cells. We found that enrichment of P300 at the *FASN* promoter was more than 10-time higher than non-specific IgG (Figure 1B), indicating that P300 binds to the *FASN* gene promoter in PCa cells.

Because P300 primarily functions as a histone acetyltransferase, we sought to assess the enrichment of H3K27Ac in the *FASN* promoter using ChIP assays. We found that H3K27Ac was highly enriched in the *FASN* promoter in LNCaP cells (Figure 1C). Importantly, knockdown of endogenous P300 substantially decreased H3K27Ac levels in the *FASN* promoter as well as global H3K27Ac levels (Figure 1C). The effectiveness of knockdown of P300 protein was evident in western blot analysis (Figure 1C). These data suggest that P300 plays an important role in mediating H3K27Ac in the *FASN* promoter. In agreement with this observation, treatment of LNCaP cells with panobinostat (LBH589), a pan histone deacetylase (HDAC) inhibitor, not only increased the global level of H3H27Ac, but also significantly increased H3K27Ac levels in the *FASN* promoter, and this effect was not due to increased expression of P300 protein (Figure 1D). These data suggest that P300 functions as an important regulator of the *FASN* gene by binding to and increasing H3K27Ac levels in the promoter.

### P300 regulates FASN mRNA and protein expression in PCa cells

**Figure 2:**
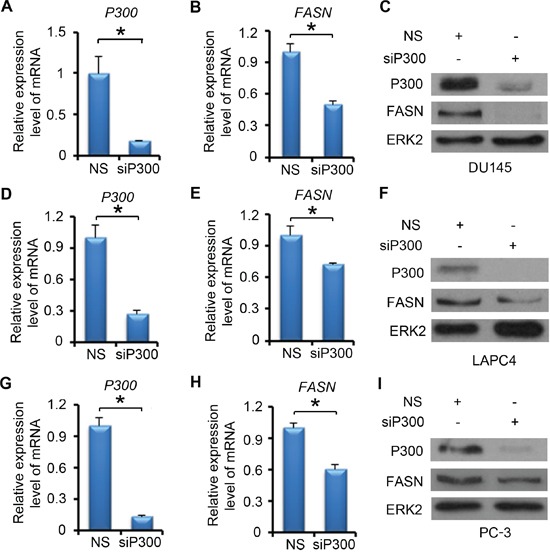
P300 regulates the expression of FASN mRNA and protein DU145 **A-C.** LAPC4 **D-F.** and PC-3 **G-I.** cells were transfected with non-specific (NS) control or P300-specific siRNAs. 48 h after transfection, mRNA of P300 and FASN (A-B, D-E and G-H) were measured by real-time PCR and protein expression of P300, FASN and ERK2 (loading control) were analyzed by western blot (C, F and I). Columns, mean values among three replicates; error bars, SD. * *P* < 0.05.

We next examined the effect of P300 on FASN expression at both mRNA and protein levels. DU145 cells were treated with non-specific (NS) control or P300-specific siRNA. We demonstrated that P300 was effectively knocked down at both mRNA and protein levels and that knockdown of P300 largely decreased FASN mRNA and protein expression in these cells (Figure 2A-2C). Similar results were obtained in other two PCa cell lines LAPC4 (Figure 2D-2F) and PC-3 (Figure 2G-2I). It is worth noting that the effect of P300 knockdown on FASN expression varied in different PCa cell lines (Figure 2C, 2F and 2I), suggesting that P300 regulation of FASN expression is likely influenced by other signaling pathways in PCa cells. Nevertheless, these data indicate that P300 regulates the expression of FASN mRNA and protein in various human PCa cell lines.

P300 contains a histone acetyltransferase (HAT) catalytic domain, which enables to promote transcription of proximal genes. The HAT activity also promotes acetylation of certain transcription factors, hence modulating the function of key transcriptional regulators [30]. Since we found that P300 binds to the *FASN* promoter and modulates H3K27Ac in that region, we sought to determine the role of the P300 HAT domain in regulation of FASN expression. We demonstrated that forced expression of wild-type P300, but not the HAT-deletion mutant (P300ΔHAT) increased expression of FASN at both mRNA and protein levels in DU145 cells (Supplementary Figure S1). These data suggest that the HAT activity is important for P300 regulation of FASN expression in PCa cells.

### The role of CBP in regulation of FASN expression in PCa cells

CBP (CREB binding protein) is a homolog of P300. These two proteins share approximately 75% sequence similarity and approximately 63% identity. Most characterized functional domains in CBP and P300 are within the highly conserved regions (CRs) [31], suggesting CBP as a potential regulator of FASN expression. To test this hypothesis, DU145 cells were transfected with non-specific (NS), CBP-specific and/or P300-specific siRNAs. After 48 h of transfection, *FASN* mRNA expression was measured by real-time PCR. We found that knocking down P300 did not affect CBP expression and vice versa (Supplementary Figure S2A and S2B). While P300 knockdown significantly decreased FASN *mRNA* expression, CBP knockdown alone only had minimal effect on FASN expression (Supplementary Figure S2C). Intriguingly, knockdown of CBP resulted in a significant decrease in FASN expression in P300-knockdown cells where P300 levels were very low (Supplementary Figure S2C). While we found that CBP enables to regulate FASN expression in cells with low levels of P300 expression, our data cannot rule out the possibility that CBP alone also plays a major role in FASN expression since western blot data showed that the effectiveness of CBP knockdown was not robust as P300 knockdown in these cells (Supplementary Figure S2D). These results are consistent with the previous findings that CBP and P300 are homologous proteins and while each has unique functions, they share many common roles in regulation of PCa cell growth and survival under both *in vitro* and *in vivo* conditions [16, 32, 33].

### P300 regulates lipid accumulation in PCa cells both *in vitro* and *in vivo*

**Figure 3:**
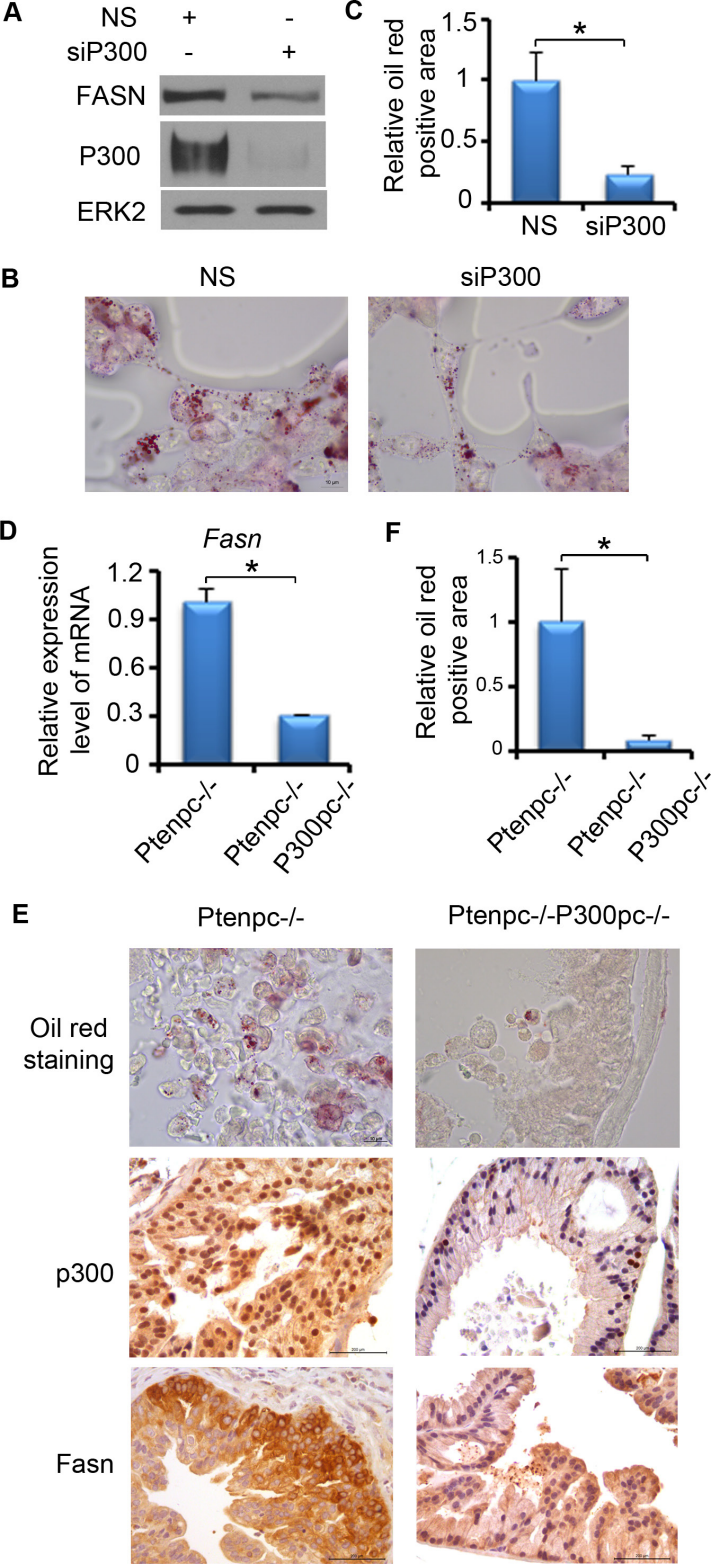
P300 affects the lipid accumulation in PCa both *in vitro* and *in vivo* **A-C.** P300 affects the lipid accumulation in PCa cells cultured *in vitro*. **A.** LNCaP cells were transfected with non-specific (NS) or P300-specific siRNAs. 72 h after transfection, cells were harvested for western blot to measure the protein expression of P300, FASN and ERK2 (loading control). **B.** LNCaP cells were transfected with non-specific (NS) or P300-specific siRNAs as in (A) and cells were subjected to oil red O staining. Scale bar, 10 μm. **C.** Quantitative data of Oil red O staining in cells shown in (B). **D-F.** P300 affects the lipid accumulation in PCa *in vivo*. **D.** RT-qPCR analysis of expression of *Fasn* mRNA in the prostate tissues from mice (n = 3) with the indicated genotypes at 4 months of age. **E.** Fresh frozen or FFPE prostate tissues from 4-month-old Pten^pc−/−^, Pten^pc−/−^;P300^pc−/−^ mice (n = 3/group) were sectioned (5 μm). Frozen sections were fixed with 4% paraformaldehyde in PBS and then followed by oil red O staining. FFPE tissues were used for p300 and Fasn IHC. Different scale bars (10 μm for oil red O staining or 200 μm for IHC) are indicated in different panels. F, Quantitative data of oil red O staining in prostate tissues shown in (E). Columns, mean values among three replicates; error bars, SD. * *P* < 0.05.

Since FASN is an important enzyme for *de novo* FA synthesis and is crucial for PCa progression, we focused on the effect of P300 on lipid accumulation in PCa cells. LNCaP cells were treated with non-specific (NS) or P300-specific siRNAs. After 72 h of treatment, we performed oil red O staining. As expected, P300 knockdown substantially inhibited FASN expression (Figure 3A); it also markedly decreased the accumulation of lipid droplets in LNCaP cells (Figure 3B and 3C). The findings in cultured cells prompted us to determine whether P300 regulates FASN expression and lipid accumulation *in vivo*. To this end, we employed prostate-specific *Pten* knockout mice as a PCa model. We demonstrated previously that knockout of p300 decreases cell proliferation in Pten-null tumors and delays tumor progression [16]. RT-qPCR analysis demonstrated that *Fasn* mRNA was readily expressed in *Pten* knockout (*Pten*^pc−/−^) mouse prostate tumors (n = 3) (Figure 3D). Importantly, *Fasn* mRNA level was significantly downregulated in *Pten* and *p300* double knockout prostatic cells (*Pten*^pc−/−^;*P300*^pc−/−^) in mice (n = 3) (Figure 3D). Immunohistochemistry (IHC) analysis showed that p300 knockout also decreased Fasn protein expression in mouse prostate tumors (Figure 3E). In line with these observations, p300 knockout also resulted in a marked decrease in accumulation of lipid droplets in *Pten* deletion tumors (Figure 3E and 3F). Thus, P300 regulates FASN expression and lipid accumulation in PCa cells under both *in vitro* and *in vivo* settings.

### Pharmacological inhibition of P300 decreases FASN expression and lipid accumulation

**Figure 4:**
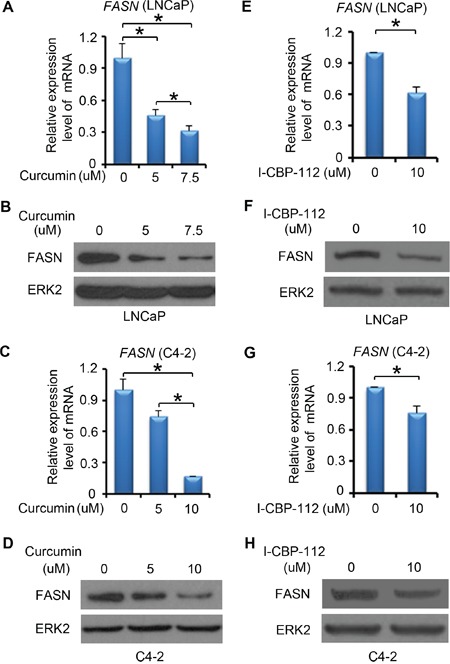
P300 inhibitors affect FASN expression **A-D.** LNCaP (A-B) and C4-2 (C-D) cells were plated in 6-well plate and after 24 h, cells were treated with DMSO or different concentrations of curcumin. After 24 h of the treatment, mRNA of *FASN* were measured by real-time PCR and protein expression levels of FASN and ERK2 (loading control) were analyzed by western blot. **E-H.** LNCaP (E-F) and C4-2 (G-H) cells were plated in 6-well plate and after 24 h, cells were treated with DMSO or I-CBP112. After 24 h of the treatment, mRNA of *FASN* was measured by real-time PCR and protein expression of FASN and ERK2 were analyzed by western blot. Columns, mean values among three replicates; error bars, SD. * *P* < 0.05.

In addition to the genetic method, we also employed a pharmacological approach to further determine the role of P300 in regulation of FASN expression and lipid metabolism. Curcumin, an active chemical in Asian spice turmeric, exhibits anticancer activity in several human cancer cell types [34, 35]. Curcumin has been identified as a potent inhibitor of P300/CBP HAT activity *in vitro* and *in vivo* [36]. LNCaP cells were treated with different concentrations of curcumin (0 - 7.5 μM) for 24 h and FASN mRNA and protein level were measured by RT-qPCR and western blot analysis, respectively. We demonstrated that curcumin treatment downregulated expression of FASN mRNA and protein in a dose-dependent manner in LNCaP cells (Figure 4A and 4B). Similar results were obtained in castration-resistant C4-2 cells following curcumin treatment (Figure 4C and 4D).

The bromodomain is an evolutionarily conserved domain in P300 and CBP. It is important for P300 and CBP to recognize specific chromatin substrates and to coordinate chromatin remodeling and transcriptional activity [37, 38]. I-CBP112 is the first highly potent and selective P300/CBP bromodomain inhibitor, which selectively binds to the P300/CBP bromodomain and inhibits the function of these proteins. We demonstrated that I-CBP112 treatment decreased FASN mRNA and protein expression in both LNCaP (Figure 4E and 4F) and C4-2 cells (Figure 4G and 4H). These data indicate that inhibition of P300 and CBP by small molecule inhibitors also decreased FASN expression in PCa cell lines.

**Figure 5:**
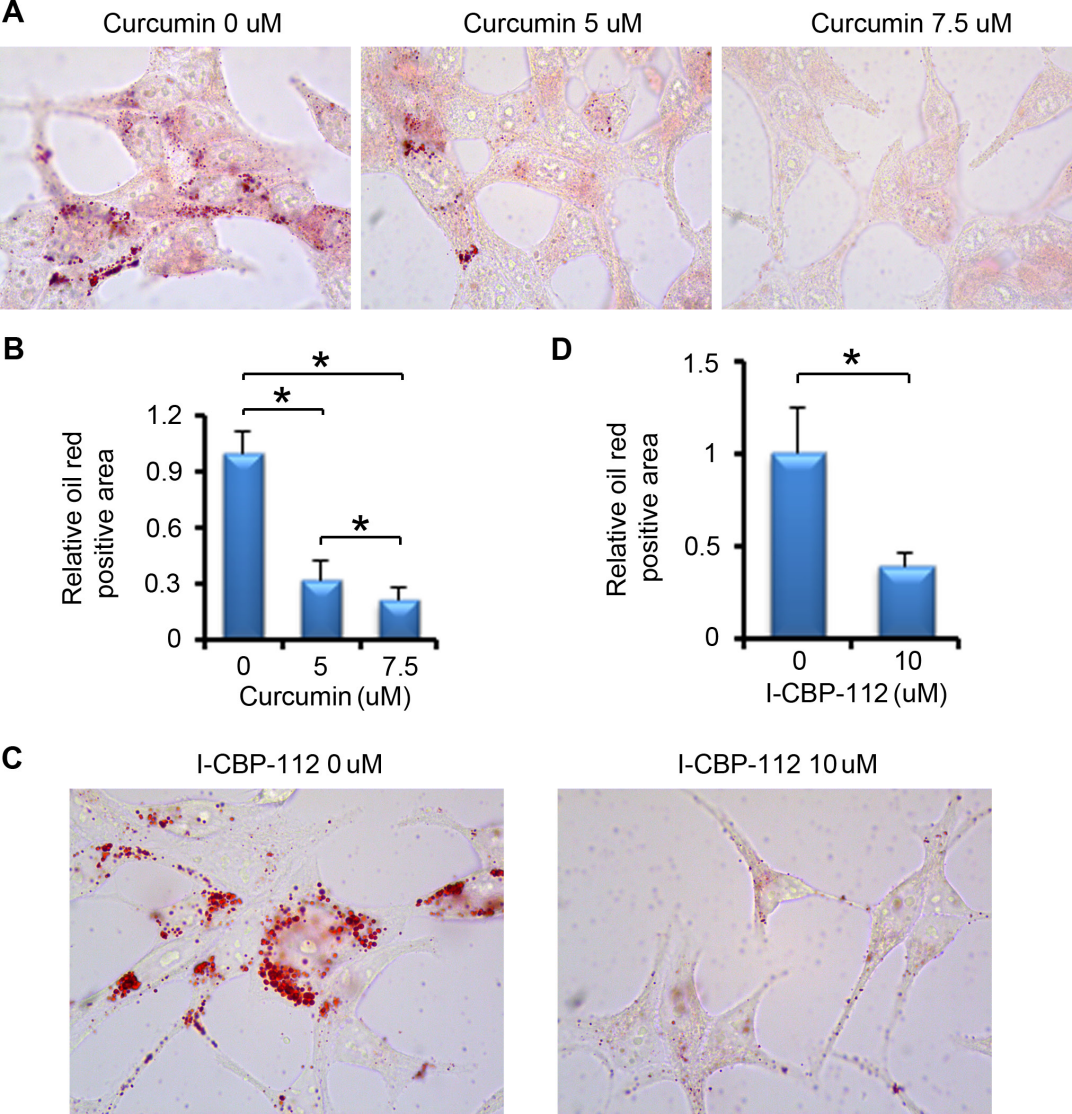
P300 inhibitors affect lipid accumulation in PCa **A.** LNCaP cells were plated in 6-well plate and after 24 h, cells were treated with DMSO or different concentrations of curcumin. After 24 h of the treatment, cells were subjected to oil red O staining. **B.** Quantitative data of oil red O staining in cells shown in (A). Columns, mean values among three replicates; error bars, SD. * *P* < 0.05. **C.** LNCaP cells were plated in 6-well plate and after 24 h, cells were treated with DMSO or I-CBP112. After 24 h of the treatment, cells were subjected to oil red O staining. **D.** Quantitative data of oil red O staining in cells shown in (C). Columns, mean values among three replicates; error bars, SD. * *P* < 0.05.

We next examined the impact of pharmacological inhibition of P300 on lipid accumulation in PCa cells. We found that curcumin treatment significantly decreased lipid droplet accumulation in a dose-dependent manner in LNCaP cells (Figure 5A and 5B). The oil red O staining also showed a significant decrease in the lipid accumulation in the cells treated with the P300/CBP bromodomain inhibitor I-CBP112 (Figure 5C and 5D). Thus, consistent with the effect on FASN expression, pharmacological inhibition of P300 and CBP also inhibits lipid accumulation in PCa cells.

### P300 and FASN expression positively correlate in human PCa specimens

**Figure 6:**
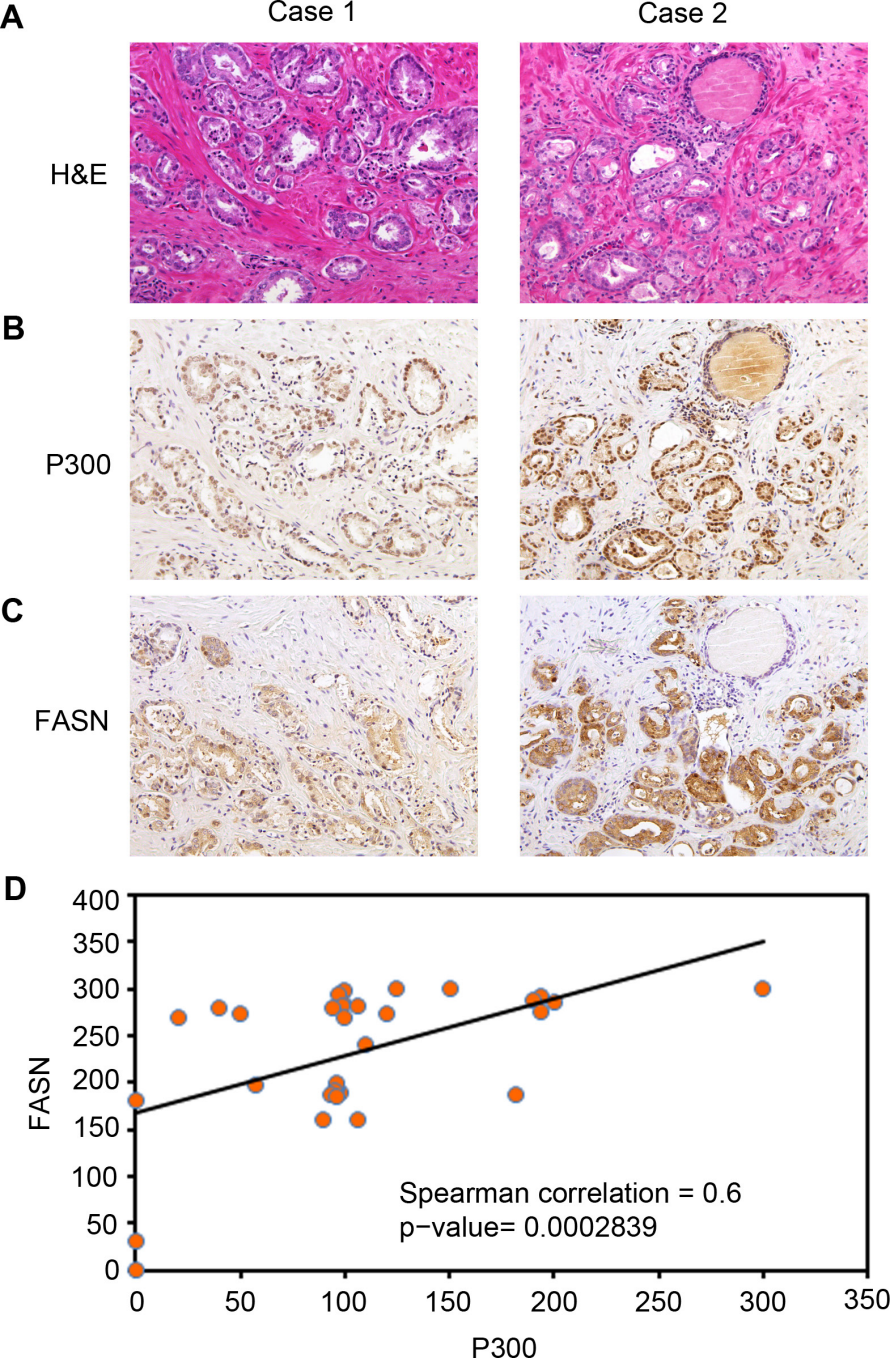
Expression of P300 positively correlates with FASN protein levels in human PCa specimens **A-C.** Representative images of H&E staining (A), P300 (B), and FASN (C) IHC in prostate cancers exhibiting low (left panels) and high (right panels) expression of P300 and FASN proteins. **D.** Correlation analysis of the staining index (SI) for expression of P300 and FASN proteins in human PCa specimens examined. Nonparameter Spearman correlation co-efficiency and the *P* values are also shown.

To gain the clinical relevance of P300 regulation of FASN expression observed in PCa cell lines, we sought to determine if P300 and FASN expression correlate in human PCa patient specimens. To this end, we examined the expression of these proteins by IHC in PCa specimens obtained from a cohort of 32 patients. Examples of both strong and weak staining of P300 and FASN proteins and hematoxylin and eosin (H&E) staining are shown in Figure 6A, 6B and 6C. The distribution of P300 and FASN protein expression in these specimens are shown in Figure 6D. Nonparametric Spearman rank correlation analysis indicates that there was a strong correlation between P300 and FASN protein expression in this cohort and the correlation is statistically significant (*r* = 0.6; *P* < 0.001). Thus, expression of P300 positively correlates with FASN protein levels in human PCa specimens, suggesting that P300 may regulate FASN expression in PCa patients.

### FASN is a key mediator of P300-induced growth of PCa cells in culture and in mice

**Figure 7:**
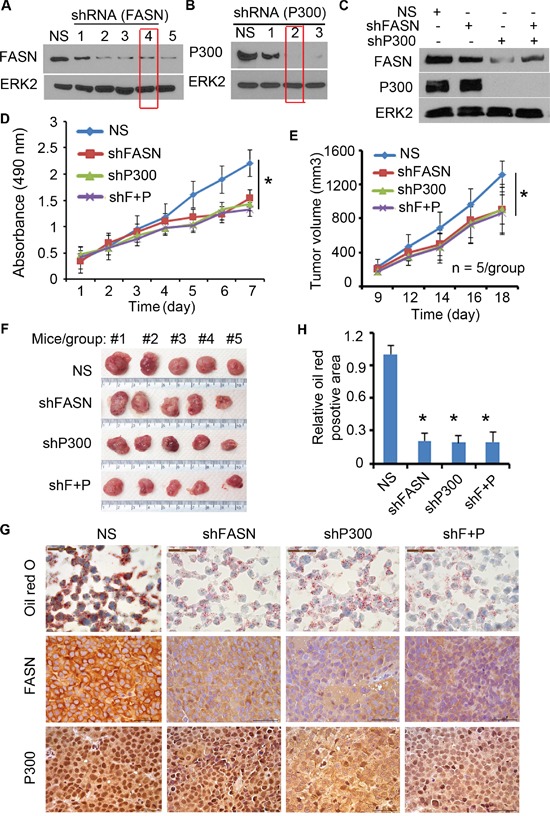
P300 regulates the growth of PCa through FASN **A-B.** C4-2 cells were infected with different FASN shRNA (A) or P300 shRNA (B) and after 72 h of infection, cells were harvested for western blot analysis of expression FASN, P300 and ERK2 (loading control) proteins. **C-D.** C4-2 cells infected with FASN shRNA and/or P300 shRNA were subjected to analysis of expression of these proteins by western blot (C) and MTS assay (D). shF+P, FASN shRNA plus P300 shRNA. * *P* < 0.05. **E-H.** C4-2 cells infected with indicated shRNAs were injected s.c. into the right flank of nude mice (n = 5/group). The volume of each tumor at each time point (E), tumors at the end of treatment (F) and oil red O staining and P300 and FASN IHC in xenografts (G and H) are shown. Columns, mean values among three replicates; error bars, SD. * *P* < 0.05.

Previous studies report that FASN expression is increased during PCa progression [11] and that P300 promotes PCa proliferation and progression [14, 16]. Next, we sought to determine the role of FASN expression in P300-mediated PCa cell growth. To this end, we first identified the most effective lentiviral FASN- and P300-specific short hairpin RNA (shRNA) (Figure 7A and 7B). Next, C4-2 cells were infected with control, FASN- and/or P300-specific shRNA and lentivirus-infected cells were used for western blot, MTS assay and animal studies. As demonstrated in Figure 7C, knockdown of both FASN and P300 was effective in C4-2 cells. MTS assay showed that knockdown of FASN or P300 alone decreased cell viability compared with the control group (Figure 7D). Importantly, no further decrease in cell viability was detected when both FASN and P300 were knocked down (Figure 7D). Similar to the results in C4-2 cells cultured *in vitro* (Figure 7D), knockdown of FASN or P300 alone inhibited growth of C4-2 xenografts in mice (Figure 7E and 7F). However, double knockdown of P300 and FASN had little or no further inhibition of C4-2 tumor growth in mice (Figure 7E and 7F). Oil red O staining in the xenograft tumor tissues showed that knockdown of P300 or FASN decreased the lipid accumulation in C4-2 xenografts *in vivo*, but double knockdown of P300 and FASN did not result in further significant inhibition of lipid accumulation in C4-2 tumors in mice (Figure 7G and 7H), consistent with the tumor growth data (Figure 7E and 7F). These data suggest that FASN is a key downstream effector that mediates P300-induced growth of PCa cells both in culture and in mice.

## DISCUSSION

Metabolic reprogramming in cancer cells has been recognized approximately a century ago. FASN is a key metabolic enzyme that plays a central role in lipid homeostasis. In agreement with the finding that lipid metabolism, rather than aerobic glycolysis appears to be the key energy source for PCa [4], increased expression of FASN correlates with worse prognosis and poor survival of PCa patients [39]. P300 is an essential transcription co-activator. Increasing evidence suggests that P300 is often overexpressed in human PCa and has been implicated in PCa proliferation and progression [16, 19]. In the present study, we identified *FASN* as a direct downstream target gene of P300 in PCa cells. We provided evidence that P300 binds to and increases H3K27Ac levels at the *FASN* promoter and that P300 expression correlates with FASN protein levels in human PCa specimens. In agreement with regulation of FASN expression, P300 also regulates lipid accumulation in PCa cells both *in vitro* and *in vivo*. Most importantly, we demonstrated FASN as a key mediator of P300-induced growth of PCa cells in culture and in mice. Thus, for the first time our findings not only unravel a previously undefined regulatory mechanism that leads to overexpression of FASN in PCa, but also uncover a molecular module that links the role of P300 to lipid metabolism in PCa.

Due to the unique energy dependence of PCa on lipid metabolism [4], it is of paramount importance to understand how the lipid metabolic pathways are deregulated in PCa. While it is well documented that FASN is often overexpressed during PCa progression, the molecular mechanisms as to how the FASN gene is regulated in PCa cells remains poorly understood. Approximately two decades ago, Swinnen and colleagues demonstrated that FASN expression is upregulated by androgens in LNCaP cells [40], an effect similar to progestins in breast cancer cells [41]. In line with this observation, further studies found that FASN expression decreases initially in LNCaP tumors following acute castration in mice [42]. However, FASN levels resume and continuously rise after long-term castration and the underlying mechanism is unclear thus far [42]. In the current study, we uncover that FASN expression is regulated by P300 in PCa cells both *in vitro* and *in vivo*, although it is warranted to determine which transcription factor(s) work in concert with P300 to promote FASN expression in these cells. Notably, it has been reported that P300 expression is upregulated following androgen deprivation treatment in LNCaP [43] and its expression is associated with human PCa progression [19]. Based upon the findings from us and others, we envision a model where P300 cooperates with the transcription factor(s) such as AR to transcriptionally regulate FASN expression in PCa cells. Following androgen ablation, P300 levels increase, which antagonizes the negative effect of androgen deprivation on FASN expression and ultimately leads to FASN upregulation in PCa after long-term castration. Further investigation of this hypothesis is warranted, findings from which could lead to define novel molecular mechanism that regulates FASN expression during castration-resistant progression of PCa, thereby providing alternative strategies for effective treatment of castration-resistant PCa in the clinic.

P300 possesses an intrinsic histone acetyltransferase (HAT) activity, which acetylates histones and other proteins. Histone acetylation remodels chromatin to relax its superstructure and makes chromosomal DNA more accessible [44]. Using ChIP assay, we demonstrated that P300 binds to the *FASN* promoter in cells. We further showed that H3K27Ac levels are readily detectable in the *FASN* promoter and knockdown of P300 substantially decreases H3K27Ac levels, suggesting P300 is a key factor responsible for H3K27Ac in the *FASN* promoter. In agreement with this observation, we found that the histone acetyltransferase (HAT) activity of P300 is required for P300 regulation of FASN expression in PCa cells. In addition to identification of the role of the HAT domain, we demonstrated that the evolutionarily conserved bromodomain is also required for P300 regulation of FASN expression in PCa cells. Specifically, we found that treatment of PCa cells with I-CBP112, the first highly potent and selective P300/CBP bromodomain inhibitor, drastically downregulates FASN expression. This effect appears to be mediated by the function of the bromodomains in both P300 and CBP proteins. Thus, mechanistically, we demonstrate that both the HAT activity and bromodomain function of P300 are required for its regulation of FASN expression in PCa cells.

Acting as an acetyltransferase, P300 can facilitate gene transcription by catalyzing acetylation of both histones and nonhistone proteins such as AR and p53. Thus, it is not surprising that the role of P300 in cancer is cellular content-dependent. Findings from the studies performed in cell culture, mouse genetic and human specimens invariably show that P300 is an oncogenic protein in PCa [16, 19, 33]. However, how P300 achieves such specificity in PCa cells has not been fully understood. As aforementioned, PCa exhibits a strong requirement for *de novo* FA synthesis [4]. The newly synthesized lipids appear to be critical to support a number of cellular processes such as cancer cell proliferation and survival [9]. In the present study, we found that expression of P300 positively correlates with FASN protein levels in a cohort of human PCa specimens. We further showed that P300 regulates FASN expression and lipid accumulation in PCa cells cultured *in vitro* and in mice. Most importantly, we identified FASN as a key mediator of P300-induced PCa cell growth *in vitro* and mice. Our data suggest that P300 functions as a unique oncogenic factor in PCa by, atleast in part, increasing FASN expression and subsequent FA synthesis and lipid metabolism. Given that *de novo* lipogenesis is a promising therapeutic target of PCa [45], the discovery of P300 regulation of FASN expression could lead to development of new strategies for PCa treatment.

## MATERIALS AND METHODS

### Cell lines, cell culture and transfection

Cell lines PC-3, DU145, LNCaP were purchased from American Type Culture Collection (Manassas, VA). C4-2 cells were purchased from Uro Corporation (Oklahoma City, OK). PC-3, DU145, LNCaP and C4-2 cells were cultured in RPMI 1640 containing 10% fetal bovine serum (FBS), 100 μg/ml streptomycin, 100 U/ml penicillin, and 0.25 μg/ml amphotericin B. LAPC4 cells were kindly provided by Dr. Charles Sawyers (Memorial Sloan-Kettering Cancer Center, New York, NY) and were maintained in Iscove's Modified Dulbecco's Media with 10% FBS. Cells were incubated at 37°C with 5% CO2. These cell lines have been tested and authenticated (karyotyping, AR expression, and PTEN mutation status) for fewer than 6 months prior to this submission. Cell transfection was performed by electroporation using an Electro Square Porator ECM 830 (BTX) as described previously [21] or lipofectamine 2,000 (Life Technologies). Approximately 75% to 90% transfection efficiencies were routinely achieved.

### Generation of prostate-specific *Pten* single- and *Pten* and *p300* double knockout mice

*Pten* conditional knockout (*Pten*^L/L^) mice were generated originally in the laboratory of Dr. Hong Wu, University of California Los Angeles [22] and purchased from The Jackson Laboratory. *P300* conditional knockout (*p300*^L/L^) mice were provided by Dr. Jan van Deursen at Mayo Clinic [23]. *Pb-Cre*4 transgenic mice were generated originally in the laboratory of Dr. Pradip Roy-Burman, University of Southern California [24] and acquired from the National Cancer Institute (NCI) Mouse Repository. As we reported previously [16], cohorts of prostate-specific *Pten* deletion (*Pten*^pc−/−^) and *Pten*/*p300* double deletion (*Pten*^pc−/−^;*p300*^pc−/−^) mice were generated from Cre^+^;*p300*^pc+/−^;*Pten*^pc+/−^ males and *p300*^L/+^;*Pten*^L/+^ females, which were obtained by cross-breeding *Pb-Cre*4 males with *p300*^L/L^ and *Pten*^L/L^ females. All procedures were approved by the Mayo Clinic Institutional Animal Care and Use Committee (IACUC) and conform to the legal mandates and federal guidelines for the care and maintenance of laboratory animals.

### Antibodies and reagents

Antibodies against P300 (C-20), CBP (C-20) and ERK2 (D-2) were purchased from Santa Cruz Biotechnology. Antibody against FASN (NB400-114) for western blot was purchased from Novosbio. FASN antibody (C20G5) for IHC staining was purchased from Cell signaling Technology. Antibody against pan histone H3 and H3K27Ac (ab4729) were purchased from Abcam. Panobinostat (LBH589) was obtained from Astar Biotech LLC (Richmond, VA). Curcumin was purchased from Sigma-Aldrich. I-CBP-112 was purchased from Xcess Biosciences.

### Western blot analysis

Protein samples were prepared by lysing cells in modified RIPA buffer [1×PBS, 1% Nonidet P-40, 0.1% sodium dodecyl sulfate, and protease inhibitor cocktail (Sigma-Aldrich)]. Lysates (50–100 μg) were separated on a 7.5% SDS-PAGE and transferred to a nitrocellulose membrane. The membrane was probed with the specific primary antibody and HRP-conjugated secondary antibody and then visualized by chemiluminescence.

### Semi-quantitative and real-time RT-PCR

Total RNA was isolated with Trizol reagent (Invitrogen, Carlsbad, CA). cDNA was synthesized using SuperScript II reverse transcriptase (Invitrogen). Quantitative real-time PCR was carried out using the iQ SYBR Green Supermix and an iCycler iQTM detection system (Bio-Rad) according to manufacturer's instructions. The 2−ΔΔCt method was used to calculate the relative expression level by normalizing to glyceraldehyde-3-phosphate dehydrogenase (*GAPDH*) levels. The following primer sequences were used: *P300*, forward 5′-CAATGAGATCCAAGGGGAGA-3′ and reverse 5′-ATGCATCTTTCTTCCGCACT-3′; *FASN*, forward 5′-TGAATCACCAGTGCCAACTCAAGG-3′ and reverse 5′-TCGTGTCTGAGGACTTTCAGCTTC-3′; *CBP*, forward 5′-GTGCTGGCTGAGACCCTAAC-3′ and reverse 5′-GGCTGTCCAAATGGACTTGT-3′; *Fasn*, forward 5′-TGTGCTCCCAGCATGCAGGCC-3′ and reverse 5′-GCCCGGTAGCTCTGGGTGTA-3′; *GAPDH*, forward 5′-ACCCACTCCTCCACCTTTGAC-3′ and reverse 5′-TGTTGCTGTAGCCAAATTCGTT-3′; *Gapdh*, forward 5′-ACAACTTTGGCATTGTGGAA-3′ and reverse 5′-GATGCAGGGATGATGTTCTG-3′.

### Chromatin immunoprecipitation (ChIP) assay

The ChIP assay was performed as described previously [25]. The soluble chromatin was incubated with 2 μg of non-specific control rabbit IgG, P300 or H3K27Ac antibodies. PCR was performed using primers specific for the P300 binding region in the *FASN* promoter, forward 5′-GGCTGCTCGTACCTGGTG-3′ and reverse 5′-GATGGCCGCGGTTTAAATA-3′. Quantitative real-time PCR was performed with ChIP samples using the iQ SYBR Green Supermix and an iCycler iQTM detection system (Bio-Rad) according to manufacturer's instructions.

### Oil red O staining

LNCaP cells were transfected with non-specific (NS) control or P300-specific siRNAs, or treated with curcumin or I-CBP-112 with different concentrations. After 48 h of transfection or 24 h of drug treatment, cultures were washed with 1×PBS and fixed for 1 h in a 5% formaldehyde solution in 1×PBS. After washing with 1×PBS, cells were stained with oil red O as described previously [26]. Fresh frozen prostate tissues from 4-month-old *Pten*^pc−/−^ and *P300*^pc−/−^;*Pten*^pc−/−^ mice (n = 3/group) and xenograft tumors were sectioned (5 μm). Frozen sections were fixed with 4% paraformaldehyde in 1×PBS for 1 h and then followed by oil red O staining as described above. Staining was assessed using bright-field microscopy. The lipid accumulation was quantified by measuring the oil red staining positive areas using the software Image J. Staining positive cells were scored in average six random visual fields.

### MTS assay

C4-2 cells were transfected with non-specific (NS) control or P300 and/or FASN-specific siRNAs and then seeded in 96-well plates at a density of 3,000 cells per well in 10% CSS medium. The viable cells were assessed by MTS (PRG3581; Promega, Madison, WI) assay. Absorbance values (490 nm) were measured on six repeat samples using a VERSAmax Microplate Reader.

### Human PCa specimens and IHC scoring

Thirty-two prostate cancer tissues [Gleason score (GS10), 1 case; GS9, 2 cases; GS8, 5 cases; GS7 14 cases; and GS6, 10 cases] were selected randomly from patients with biopsy-proven PCa that have been treated at the Mayo Clinic by radical retropubic prostatectomy between January 1995 and December 1998 without neoadjuvant therapy. The age of the patients ranged from 47 to 73 years (mean 62.9 years old). The study was approved by the Mayo Clinic Institutional Review Board. Antigen retrieval and immunostaining was performed as described [27, 28]. Primary antibodies used were: P300 (1:1,000) and FASN (1:100) was performed as described previously [16]. Staining intensity of each slide was graded accordingly (intensity: 0, no staining; 1, low staining; 2, media staining; and 3, strong staining). A final staining index (SI) SI score for each staining was obtained by multiplying values obtained from staining percentage and intensity and used for correlation analysis.

### Mouse xenograft studies

All mice were housed in standard conditions with a 12 h light/dark cycle and access to food and water ad libitum. The mouse studies were approved by the Mayo Clinic IACUC. Athymic (nu/nu) 6-week-old males (Harlan) were used for all *in vivo* experiments. C4-2 cells infected with FASN shRNA or P300 shRNA or both (in 100 μl serum free RPMI medium) were injected s.c. into the right flank (5×10^6^ cells) of mice. After xenografts reached the size around 200 mm^3^ (9 days after injection), volume of tumors were measured every day with a calliper. After 17 days of the injection, tumor grafts were harvested and embedded in OCT (optimum cutting temperature compound) or formalin-fixed and paraffin-embedded (FFPE). OCT and FFPE-processed tissues were subjected to oil red staining and P300 and FASN IHC, respectively.

### Statistical analysis

Experiments were carried out with three or more replicates unless otherwise stated. All values were expressed as means±SD. Comparison between two mean values was made by independent-sample t-test. For analysis of correlation between P300 and FASN protein expression in human PCa specimens, nonparametric spearman rank correlation was used. *P* < 0.05 was considered statistically significant.

## SUPPLEMENTARY FIGURES AND TABLES



## References

[1] Baade PD, Youlden DR, Krnjacki LJ (2009). International epidemiology of prostate cancer: geographical distribution and secular trends. Mol Nutr Food Res.

[2] Siegel R, Ward E, Brawley O, Jemal A (2011). Cancer statistics, 2011: the impact of eliminating socioeconomic and racial disparities on premature cancer deaths. CA Cancer J Clin.

[3] Ward PS, Thompson CB (2012). Metabolic reprogramming: a cancer hallmark even warburg did not anticipate. Cancer Cell.

[4] Ackerstaff E, Pflug BR, Nelson JB, Bhujwalla ZM (2001). Detection of increased choline compounds with proton nuclear magnetic resonance spectroscopy subsequent to malignant transformation of human prostatic epithelial cells. Cancer Res.

[5] Picchio M, Messa C, Landoni C, Gianolli L, Sironi S, Brioschi M, Matarrese M, Matei DV, De Cobelli F, Del Maschio A, Rocco F, Rigatti P, Fazio F (2003). Value of [11C]choline-positron emission tomography for re-staging prostate cancer: a comparison with [18F]fluorodeoxyglucose-positron emission tomography. J Urol.

[6] Rossi S, Graner E, Febbo P, Weinstein L, Bhattacharya N, Onody T, Bubley G, Balk S, Loda M (2003). Fatty acid synthase expression defines distinct molecular signatures in prostate cancer. Mol Cancer Res.

[7] Shah US, Dhir R, Gollin SM, Chandran UR, Lewis D, Acquafondata M, Pflug BR (2006). Fatty acid synthase gene overexpression and copy number gain in prostate adenocarcinoma. Hum Pathol.

[8] Ashida S, Nakagawa H, Katagiri T, Furihata M, Iiizumi M, Anazawa Y, Tsunoda T, Takata R, Kasahara K, Miki T, Fujioka T, Shuin T, Nakamura Y (2004). Molecular features of the transition from prostatic intraepithelial neoplasia (PIN) to prostate cancer: genome-wide gene-expression profiles of prostate cancers and PINs. Cancer Res.

[9] Migita T, Ruiz S, Fornari A, Fiorentino M, Priolo C, Zadra G, Inazuka F, Grisanzio C, Palescandolo E, Shin E, Fiore C, Xie W, Kung AL, Febbo PG, Subramanian A, Mucci L (2009). Fatty acid synthase: a metabolic enzyme and candidate oncogene in prostate cancer. J Natl Cancer Inst.

[10] Heemers HV, Verhoeven G, Swinnen JV (2006). Androgen activation of the sterol regulatory element-binding protein pathway: Current insights. Mol Endocrinol.

[11] Hamada S, Horiguchi A, Kuroda K, Ito K, Asano T, Miyai K, Iwaya K (2014). Increased fatty acid synthase expression in prostate biopsy cores predicts higher Gleason score in radical prostatectomy specimen. BMC Clin Pathol.

[12] De Schrijver E, Brusselmans K, Heyns W, Verhoeven G, Swinnen JV (2003). RNA interference-mediated silencing of the fatty acid synthase gene attenuates growth and induces morphological changes and apoptosis of LNCaP prostate cancer cells. Cancer Res.

[13] Pizer ES, Thupari J, Han WF, Pinn ML, Chrest FJ, Frehywot GL, Townsend CA, Kuhajda FP (2000). Malonyl-coenzyme-A is a potential mediator of cytotoxicity induced by fatty-acid synthase inhibition in human breast cancer cells and xenografts. Cancer Res.

[14] Vervoorts J, Luscher-Firzlaff JM, Rottmann S, Lilischkis R, Walsemann G, Dohmann K, Austen M, Luscher B (2003). Stimulation of c-MYC transcriptional activity and acetylation by recruitment of the cofactor CBP. EMBO Rep.

[15] Solt I, Magyar C, Simon I, Tompa P, Fuxreiter M (2006). Phosphorylation-induced transient intrinsic structure in the kinase-inducible domain of CREB facilitates its recognition by the KIX domain of CBP. Proteins.

[16] Zhong J, Ding L, Bohrer LR, Pan Y, Liu P, Zhang J, Sebo TJ, Karnes RJ, Tindall DJ, van Deursen J, Huang H (2014). p300 acetyltransferase regulates androgen receptor degradation and PTEN-deficient prostate tumorigenesis. Cancer Res.

[17] Teufel DP, Freund SM, Bycroft M, Fersht AR (2007). Four domains of p300 each bind tightly to a sequence spanning both transactivation subdomains of p53. Proc Natl Acad Sci U S A.

[18] Pao GM, Janknecht R, Ruffner H, Hunter T, Verma IM (2000). CBP/p300 interact with and function as transcriptional coactivators of BRCA1. Proc Natl Acad Sci U S A.

[19] Debes JD, Sebo TJ, Lohse CM, Murphy LM, Haugen DA, Tindall DJ (2003). p300 in prostate cancer proliferation and progression. Cancer Res.

[20] Ianculescu I, Wu DY, Siegmund KD, Stallcup MR (2012). Selective roles for cAMP response element-binding protein binding protein and p300 protein as coregulators for androgen-regulated gene expression in advanced prostate cancer cells. J Biol Chem.

[21] Barski A, Cuddapah S, Cui K, Roh TY, Schones DE, Wang Z, Wei G, Chepelev I, Zhao K (2007). High-resolution profiling of histone methylations in the human genome. Cell.

[22] Kalkhoven E (2004). CBP and p300: HATs for different occasions. Biochem Pharmacol.

[23] Wang F, Marshall CB, Ikura M (2013). Transcriptional/epigenetic regulator CBP/p300 in tumorigenesis: structural and functional versatility in target recognition. Cell Mol Life Sci.

[24] Ding L, Chen S, Liu P, Pan Y, Zhong J, Regan KM, Wang L, Yu C, Rizzardi A, Cheng L, Zhang J, Schmechel SC, Cheville JC, Van Deursen J, Tindall DJ, Huang H (2014). CBP Loss Cooperates with PTEN Haploinsufficiency to Drive Prostate Cancer: Implications for Epigenetic Therapy. Cancer Res.

[25] Santer FR, Hoschele PP, Oh SJ, Erb HH, Bouchal J, Cavarretta IT, Parson W, Meyers DJ, Cole PA, Culig Z (2011). Inhibition of the acetyltransferases p300 and CBP reveals a targetable function for p300 in the survival and invasion pathways of prostate cancer cell lines. Mol Cancer Ther.

[26] Li M, Zhang Z, Hill DL, Wang H, Zhang R (2007). Curcumin, a dietary component, has anticancer, chemosensitization, and radiosensitization effects by down-regulating the MDM2 oncogene through the PI3K/mTOR/ETS2 pathway. Cancer Res.

[27] Bao B, Ali S, Banerjee S, Wang Z, Logna F, Azmi AS, Kong D, Ahmad A, Li Y, Padhye S, Sarkar FH (2012). Curcumin analogue CDF inhibits pancreatic tumor growth by switching on suppressor microRNAs and attenuating EZH2 expression. Cancer Res.

[28] Balasubramanyam K, Varier RA, Altaf M, Swaminathan V, Siddappa NB, Ranga U, Kundu TK (2004). Curcumin, a novel p300/CREB-binding protein-specific inhibitor of acetyltransferase, represses the acetylation of histone/nonhistone proteins and histone acetyltransferase-dependent chromatin transcription. J Biol Chem.

[29] Zeng L, Zhang Q, Gerona-Navarro G, Moshkina N, Zhou MM (2008). Structural basis of site-specific histone recognition by the bromodomains of human coactivators PCAF and CBP/p300. Structure.

[30] Mujtaba S, He Y, Zeng L, Yan S, Plotnikova O, Sachchidanand Sanchez R, Zeleznik-Le NJ, Ronai Z, Zhou MM (2004). Structural mechanism of the bromodomain of the coactivator CBP in p53 transcriptional activation. Mol Cell.

[31] Bandyopadhyay S, Pai SK, Watabe M, Gross SC, Hirota S, Hosobe S, Tsukada T, Miura K, Saito K, Markwell SJ, Wang Y, Huggenvik J, Pauza ME, Iiizumi M, Watabe K (2005). FAS expression inversely correlates with PTEN level in prostate cancer and a PI 3-kinase inhibitor synergizes with FAS siRNA to induce apoptosis. Oncogene.

[32] Swinnen JV, Esquenet M, Goossens K, Heyns W, Verhoeven G (1997). Androgens stimulate fatty acid synthase in the human prostate cancer cell line LNCaP. Cancer Res.

[33] Chalbos D, Chambon M, Ailhaud G, Rochefort H (1987). Fatty acid synthetase and its mRNA are induced by progestins in breast cancer cells. J Biol Chem.

[34] Ettinger SL, Sobel R, Whitmore TG, Akbari M, Bradley DR, Gleave ME, Nelson CC (2004). Dysregulation of sterol response element-binding proteins and downstream effectors in prostate cancer during progression to androgen independence. Cancer Res.

[35] Heemers HV, Schmidt LJ, Kidd E, Raclaw KA, Regan KM, Tindall DJ (2010). Differential regulation of steroid nuclear receptor coregulator expression between normal and neoplastic prostate epithelial cells. Prostate.

[36] Gorisch SM, Wachsmuth M, Toth KF, Lichter P, Rippe K (2005). Histone acetylation increases chromatin accessibility. J Cell Sci.

[37] Sadowski MC, Pouwer RH, Gunter JH, Lubik AA, Quinn RJ, Nelson CC (2014). The fatty acid synthase inhibitor triclosan: repurposing an anti-microbial agent for targeting prostate cancer. Oncotarget.

[38] Chen S, Bohrer LR, Rai AN, Pan Y, Gan L, Zhou X, Bagchi A, Simon JA, Huang H (2010). Cyclin-dependent kinases regulate epigenetic gene silencing through phosphorylation of EZH2. Nat Cell Biol.

[39] Wang S, Gao J, Lei Q, Rozengurt N, Pritchard C, Jiao J, Thomas GV, Li G, Roy-Burman P, Nelson PS, Liu X, Wu H (2003). Prostate-specific deletion of the murine Pten tumor suppressor gene leads to metastatic prostate cancer. Cancer Cell.

[40] Kasper LH, Fukuyama T, Biesen MA, Boussouar F, Tong C, de Pauw A, Murray PJ, van Deursen JM, Brindle PK (2006). Conditional knockout mice reveal distinct functions for the global transcriptional coactivators CBP and p300 in T-cell development. Mol Cell Biol.

[41] Wu X, Wu J, Huang J, Powell WC, Zhang J, Matusik RJ, Sangiorgi FO, Maxson RE, Sucov HM, Roy-Burman P (2001). Generation of a prostate epithelial cell-specific Cre transgenic mouse model for tissue-specific gene ablation. Mech Dev.

[42] Huang H, Zegarra-Moro OL, Benson D, Tindall DJ (2004). Androgens repress Bcl-2 expression via activation of the retinoblastoma (RB) protein in prostate cancer cells. Oncogene.

[43] Swinnen JV, Van Veldhoven PP, Esquenet M, Heyns W, Verhoeven G (1996). Androgens markedly stimulate the accumulation of neutral lipids in the human prostatic adenocarcinoma cell line LNCaP. Endocrinology.

[44] Linja MJ, Porkka KP, Kang Z, Savinainen KJ, Janne OA, Tammela TL, Vessella RL, Palvimo JJ, Visakorpi T (2004). Expression of androgen receptor coregulators in prostate cancer. Clin Cancer Res.

[45] Comuzzi B, Lambrinidis L, Rogatsch H, Godoy-Tundidor S, Knezevic N, Krhen I, Marekovic Z, Bartsch G, Klocker H, Hobisch A, Culig Z (2003). The transcriptional co-activator cAMP response element-binding protein-binding protein is expressed in prostate cancer and enhances androgen- and anti-androgen-induced androgen receptor function. Am J Pathol.

[46] Wang D, Garcia-Bassets I, Benner C, Li W, Su X, Zhou Y, Qiu J, Liu W, Kaikkonen MU, Ohgi KA, Glass CK, Rosenfeld MG, Fu XD (2011). Reprogramming transcription by distinct classes of enhancers functionally defined by eRNA. Nature.

