# Supplementary material for: P300 acetyltransferase regulates fatty acid synthase expression, lipid metabolism and prostate cancer growth
Source: Oncotarget. 2016 Feb 25;7(12):15135–49. doi: 10.18632/oncotarget.7715 (PMC4924775; doi:10.18632/oncotarget.7715)
Supplement: Supplementary file 1 [file oncotarget-07-15135-s001.pdf]

SUPPLEMENTARY FIGURES

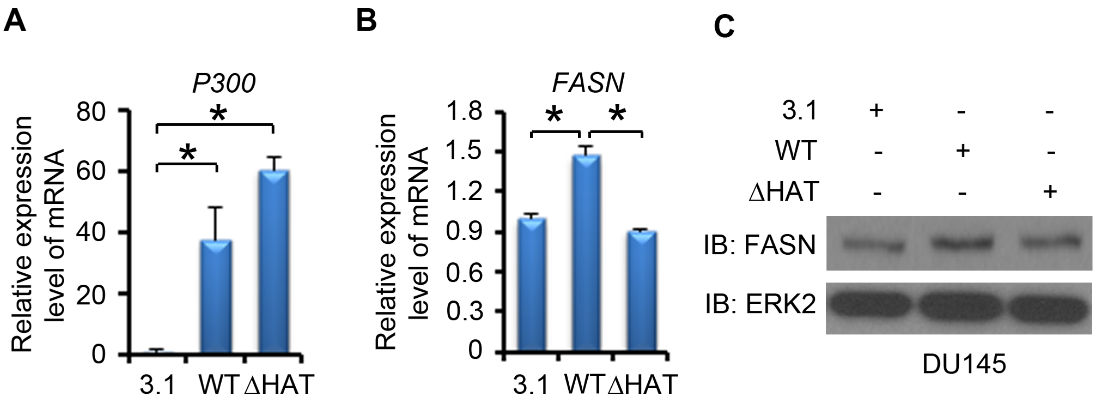

Supplementary Figure S1: The role of the HAT domain in P300 in regulation of FASN expression in PCa cells. DU145 cells were transfected with empty vector, wild-type (WT) P300 and the HAT deletion mutant (ΔHAT). After 48 h of transfection, mRNA of P300 and FASN were measured by real-time PCR and protein expression of FASN and ERK2 (loading control) were analyzed by western blot. Columns, mean values among three replicates; error bars, SD. * $P < 0.05$.

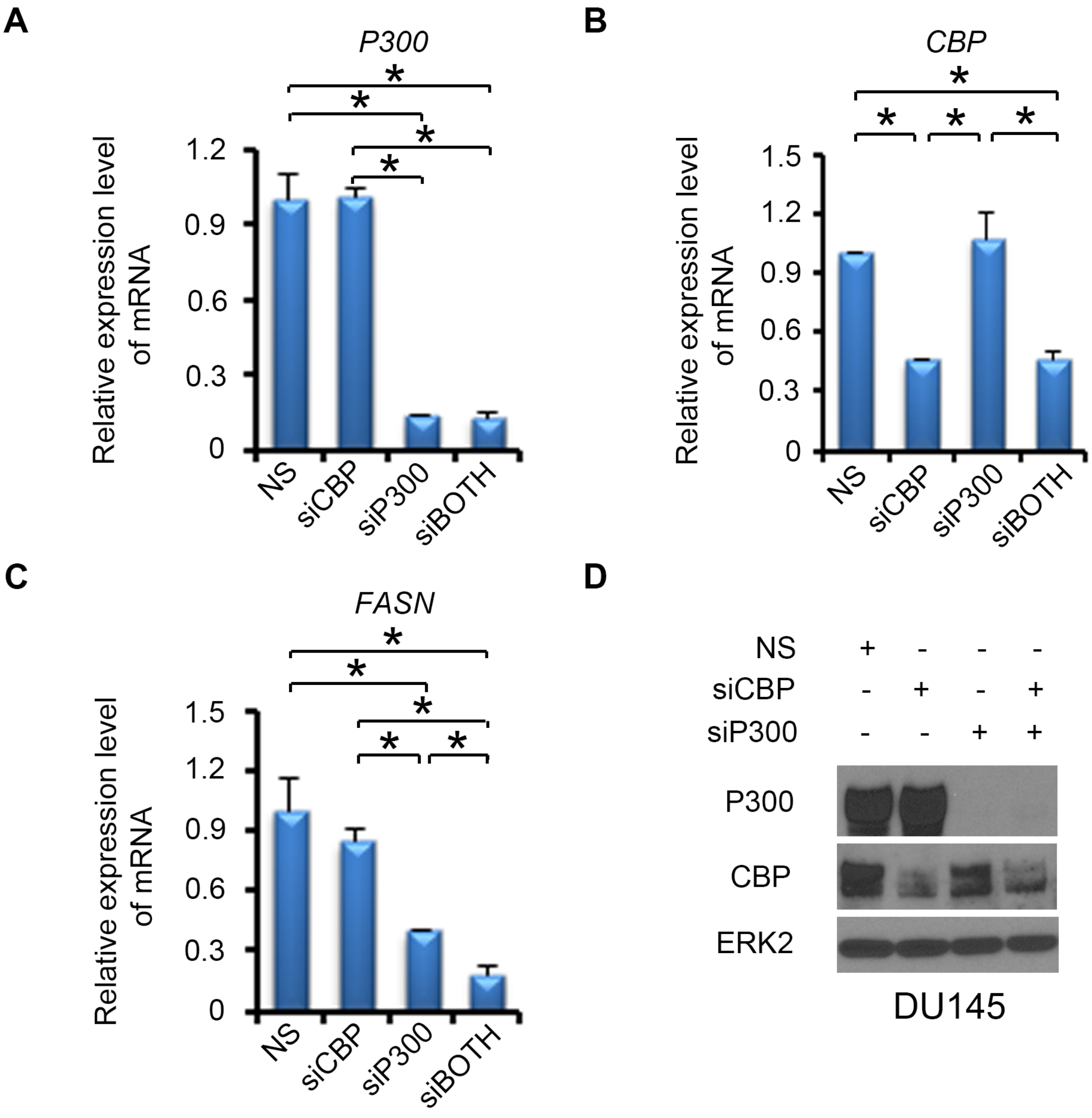

Supplementary Figure S2: The role of CBP in regulation of FASN expression in PCa cells. DU145 cells were transfected with non-specific (NS) control or CBP- and/or P300-specific siRNA. After 48 h of transfection, mRNA of P300, CBP and FASN were measured by real-time PCR and expression of P300, CBP and ERK2 proteins (loading control) were analyzed by western blot. Columns, mean values among three replicates; error bars, SD. * $P < 0.05$.
